# Development and Validation of an Interleukin-6 Nomogram to Predict Primary Non-response to Infliximab in Crohn’s Disease Patients

**DOI:** 10.3389/fphar.2021.654985

**Published:** 2021-04-14

**Authors:** Yueying Chen, Hanyang Li, Qi Feng, Jun Shen

**Affiliations:** ^1^ Division of Gastroenterology and Hepatology, Key Laboratory of Gastroenterology and Hepatology, Ministry of Health, Inflammatory Bowel Disease Research Center, Renji Hospital, School of Medicine, Shanghai Jiao Tong University, Shanghai Institute of Digestive Disease, Shanghai, China; ^2^ Department of Radiology, Renji Hospital, School of Medicine, Shanghai Jiao Tong University, Shanghai, China

**Keywords:** interleukin-6, primary non-response, crohn’s disease, infliximab, bioinfomatics

## Abstract

**Background:** The primary non-response (PNR) rate of infliximab (IFX) varies from 20 to 46% for the treatment of Crohn’s disease (CD). Detected PNR reduces the improper use of specific treatments. To date, there is hardly any knowledge regarding early markers of PNR. The aim of this study was to evaluate the role of Interleukin-6 (IL-6) as an early predictor of PNR of IFX for the treatment of CD.

**Methods:** We enrolled 322 bio-naïve patients diagnosed with CD from January 2016 to May 2020. Primary response was determined at week 14. Multivariable logistic regression was used to construct prediction models. Area under the curve (AUC), calibration and decision curve analyses (DCA) were assessed in the validation cohort. GEO data were analyzed to identify potential mechanisms of *IL-6* in IFX therapy for CD.

**Results:** PNR occurred in 31.06% (100 of 322) patients who were assessable at week 14. IL-6 levels significantly decreased after IFX therapy (*p* < 0.001). The validation model containing IL-6 presented enhanced discrimination with an AUC of 0.908 and high calibration. Decision curve analysis (DCA) indicated that the model added extra predictive value. GEO data confirmed the IL-6 levels were increased in the PNR group and IL-6-related differentially expressed genes (DEGs) were enriched in the inflammatory response.

**Conclusions:** We concluded that IL-6 may be used as a predictive factor to assess the risk of PNR to IFX therapy.

## Introduction

Crohn’s disease (CD) is a chronic inflammatory disease with a relapsing history. The incidence and prevalence of CD have both increased worldwide and this disease has gradually become a more severe socioeconomic burden (Ng et al., 2013). Recently, the treatment of CD entered a new era. Anti-tumor necrosis factor (anti-TNF) therapy has been the first-line therapy for treating CD according to ECCO guidelines (Torres et al., 2020). Infliximab (IFX) is the most widely used anti-TNF agent that promotes mucosal healing and changes the natural history of the disease (Singh et al., 2018). However, there are still 20–46% of CD patients that show primary non-response (PNR) to IFX (Roda et al., 2016; Yokoyama et al., 2016). Leapfrogging IFX in PNR patients to other medications such as anti-IL-12/23 monoclonal antibodies or anti-leucocyte adhesion molecules avoid ineffective processes of IFX and saves medical resources. Therefore, developing a model to predict PNR to IFX in CD patients will help doctors make better decisions for CD patients.

Previous studies have proposed several factors to predict the efficacy of IFX in CD, including age, BMI, previous surgery history (Billiet et al., 2015), disease behavior (Sprakes et al., 2012), disease duration (Matsuoka et al., 2018), and pro-inflammatory biomarkers (Billiet et al., 2017). Despite evidence, a clinical prediction model is still lacking to assess the possibility of PNR prior to IFX administration based on clinical and biochemical markers. As a chronic inflammatory disease, the regulation of cytokines is associated with the pathogenesis and progression of CD (Chen and Sundrud, 2016). IL-6 is a proinflammatory factor that can exacerbate inflammation by promoting the survival of T cells and the secretion of other cytokines (Hunter and Jones, 2015). *IL-6* levels were shown to be elevated in CD patients compared to healthy individuals (Engel et al., 2015). A randomized trial showed that an anti-IL-6 antibody promoted a clinical response and clinical remission in CD patients (Danese et al., 2019). Furthermore, IL-6 served as a predictor of PNR to anti-TNF treatment in CD and patients failing anti-TNF therapy showed increased expression of IL-6 and persistent IL-6 pathway activity (Leal et al., 2015; Soendergaard et al., 2018). A prospective, multicenter study confirmed the predictive roles of IL-6 in patients treated with anti-TNF therapy (Bertani et al., 2020a). Additionally, the single nucleotide polymorphisms of IL-6 may be a promising tool for identifying CD patient response to IFX therapy (Salvador-Martín et al., 2019).

To promote individualized treatment in CD patients, it is crucial to identify predictors to estimate PNR to IFX. Different IL-6 levels present before treatment may contribute to the contrasting response to IFX therapy. Detecting PNR reduces inaccurate treatments while a predictive model determining PNR to IFX in bio-naive CD is lacking. Therefore, we aimed to identify the predictive value of IL-6 in IFX therapy and to construct a model predicting PNR in bio-naive CD patients based on clinical information and IL-6 levels.

## Methods

### Patients and Samples

A retrospective and single-center study was performed for CD cases in the Division of Gastroenterology and Hepatology, Renji hospital. Patients enrolled in this study from January 2016 to June 2019 and from July 2019 to May 2020 were assigned to the training and the validation cohorts, respectively. CD was diagnosed based on the ECCO consensus (Maaser et al., 2019). Bio-naïve patients were included and induced with 5 mg/kg of IFX (Janssen Pharmaceutical Ltd, United States). Baseline characteristics were collected prior to treatment.

Levels of biochemical indicators, including IL-6, albumin, hemoglobin, platelets, erythrocyte sedimentation rate (ESR) and CRP were obtained from previous blood analysis, and blood samples were collected for each participant before and after IFX therapy. The Westergren method and nephelometry were used to measure CRP and ESR levels. Enzyme Linked Immunosorbent Assay (ELISA) was used to detect IL-6 levels using a DPC IMMULITE 1000 system of Siemens, and the average CV value about 8.33%. The Clinical Laboratory Department (Renji Hospital, School of Medicine, Shanghai Jiao Tong University, China) performed an analysis of each sample in duplicate. This study was approved by the IRB of Shanghai Jiaotong University School of Medicine, Renji Hospital Ethics Committee (KY2020–115).

### Outcomes and Definitions

Our study defined the primary outcome as the proportion of response to IFX, which was determined when achieving clinical response or remission at week 14 (after three IFX injections and before the fourth) using an MDT (multi-disciplinary team) of experienced experts combined with endoscopic and radiological examinations (Hanauer et al., 2002; Wong and Cross, 2017). PNR was defined as: 1) failure to achieve clinical response or clinical remission, clinical response and clinical remission have been defined as a decrease in Harvey Bradshaw indices (HBI) ≥ 2, and total HBI ≤ 4, respectively (Sprakes et al., 2012). 2) need for treatment modification (discontinuation, escalation or surgery) (Roda et al., 2016; Bar-Yoseph et al., 2018; Beltrán et al., 2019).

### Statistical Analysis

Results for continuous variables were represented as mean (SDs) or median (interquartile ranges [IQRs]). Categorical variables were shown as proportions. Univariate logistic regression was applied to analyze the relationships between different factors and PNR to IFX therapy. A Chi-square test was used to compare categorical variables. The *t* test or Mann-Whitney *U* test were used to compare continuous variables.

Multivariable regression models with forward stepwise likelihood ratio algorithms were used to develop models predicting the response to infliximab 5 mg/kg through 14 weeks. Akaike information criterion (AIC) was performed to assessed the goodness of fit of two models. Discrimination of the prediction models was evaluated by receiver operating characteristics (ROC) analysis and presented as area under the curve (AUC). Odds ratios (OR) having 95% confidence intervals (CI) of final predictors were calculated. Calibration curves were used to assess the calibration of nomograms by comparing the predicted and observed probabilities. The clinical effectiveness of the models was assessed using decision curve analysis (DCA). Integrated Discrimination Improvement (IDI) was a reclassification measures showed the difference in discrimination slopes of two models, and was used to assess the improvement of risk differences between cases and non-cases (Pencina et al., 2008; Steyerberg et al., 2010; Kerr et al., 2011). All statistical analyses were performed using SPSS 25.0 and R 3.6.3 with a statistical significance of *p* < 0.05.

### Bioinformatic Analysis

#### Data Source

A gene expression microarray dataset (GSE111761) of Schmitt’s study from the GEO database was selected (Schmitt et al., 2019). GSE111761 contained three samples from anti-TNF non-responders and three from responders. The patients were diagnosed as CD and defined as responders or non-responders when they had ongoing anti-TNF therapy for over 3 months. The GSE111761 dataset was available on the GPL13497 platform (Agilent-026652 Whole Human Genome Microarray 4 × 44K v2).

#### Identification of Differentially Expressed Genes (DEGs)

R software (version 3.6.3) and the limma package in Bioconductor were used to detect DEGs in GSE111761 (Ritchie et al., 2015). DEGs were identified using selection criteria of an adjusted *p* value < 0.05 and |logFC|>1.0.

#### Enrichment Analysis

Gene Set Enrichment Analysis (GSEA) was performed using the ClusterProfile package in Bioconductor with a statistical significance of *p* < 0.05 (Yu et al., 2012).

#### Protein-Protein Interaction (PPI) Network Construction

PPI network reveals the specific and unspecific interactions of proteins, and promotes to identify therapeutic target (Petta et al., 2016; Maurel et al., 2019). STRING (version 11.0), a freely accessible database that collects, scores and integrates data, was used to predict functional relationships between proteins (Szklarczyk et al., 2019). A PPI network of genes with a score > 0.4 in STRING was constructed using Cytoscape software (version 3.7.2) (Smoot et al., 2011). The degree of protein nodes was calculated using the Cytoscape plugin CytoHubba to identify hub genes (Chin et al., 2014). Hub genes were selected with a score ≥ 4.5 based on the EPC algorithm.

#### Construction of Regulatory Network

The network of genes and their corresponding miRNAs and lncRNAs was constructed using starbase, a publicly available database mainly focusing on miRNA-target interactions (Li et al., 2014). Transcription factors (TFs) were downloaded from TRRUST (http://www.grnpedia.org/trrust/), a public database for predicting TFs of various genes through DNA sequences (Farre et al., 2003). These tools were combined to construct a multi-factor regulation network.

## Results

### Baseline Characteristics and Univariate Analyses

**FIGURE 1:**
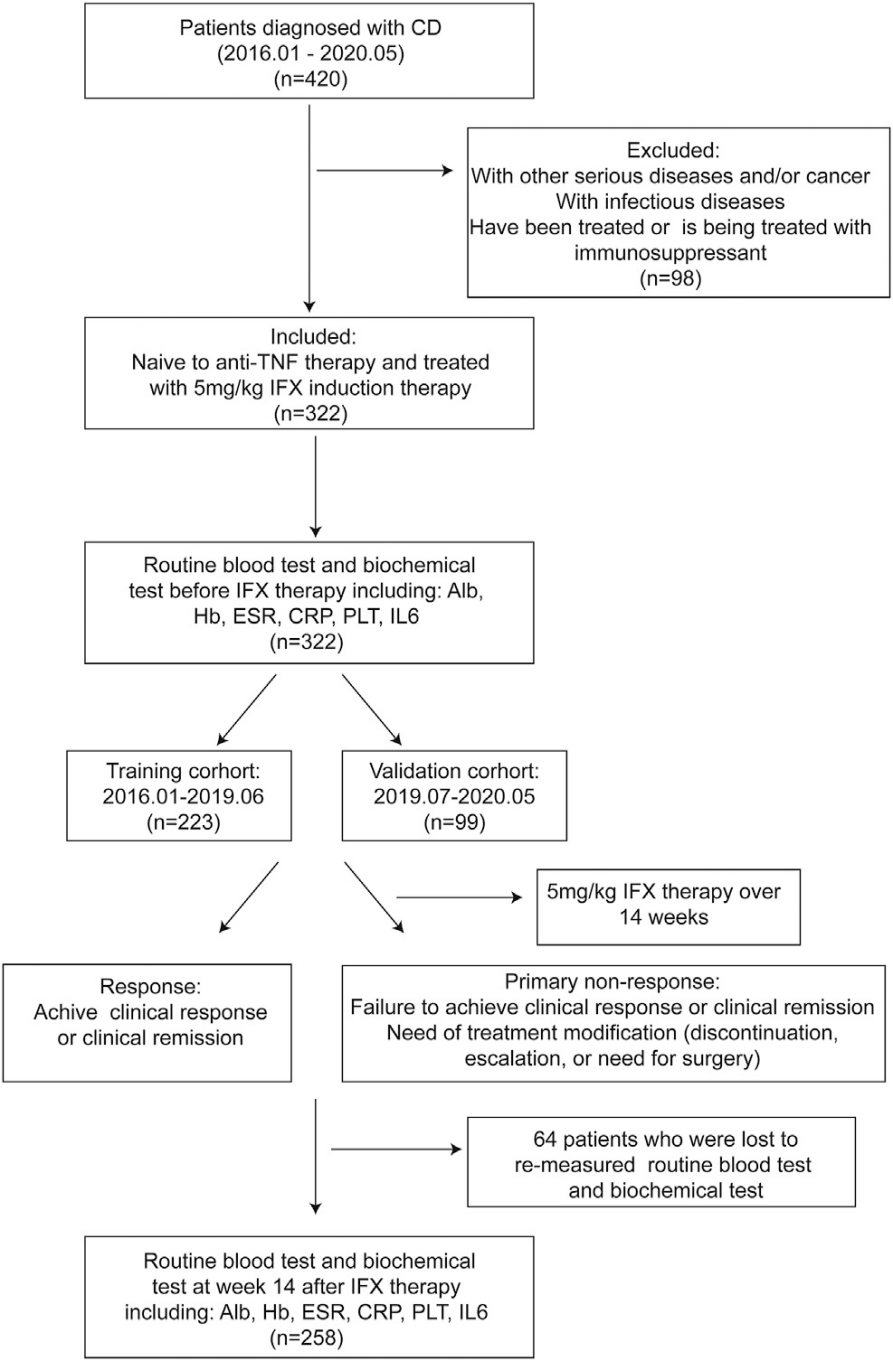
Flow diagram of the study design.

From a total of 322 active CD patients receiving 5 mg/kg IFX induction therapy, 223 and 99 were assigned to training and validation groups, respectively (Figure 1). Disease behavior was merged into two categories, including nonstricturing and nonpenetrating (B1) into one category, and stricturing and/or penetrating (B2/B3) subtypes into another category. The baseline characteristics are shown in Supplementary Table S1. The incidence of PNR in the training cohort was 30.0% (n = 67), while the incidence in the validation cohort was 33.3% (n = 33). Baseline characteristics of patients were similar between the two cohorts. Univariate regressions showed that BMI (*p* < 0.001), disease behavior (*p* < 0.001), CRP levels (*p* = 0.001), and IL-6 levels before IFX therapy (*p* = 0.002) were strongly associated with PNR to IFX treatment (Supplementary Table S2).

IL-6 levels were measured in 258 of the studied 322 patients before initiation and at week 14 before the fourth IFX injection. IL-6 levels were found to significantly decrease after IFX therapy (*p* < 0.001) and were obviously different between PNR and response groups (*p* = 0.001) (Supplementary Figure S1).

### Development of Prediction Models and Nomogram Construction

**FIGURE 2:**
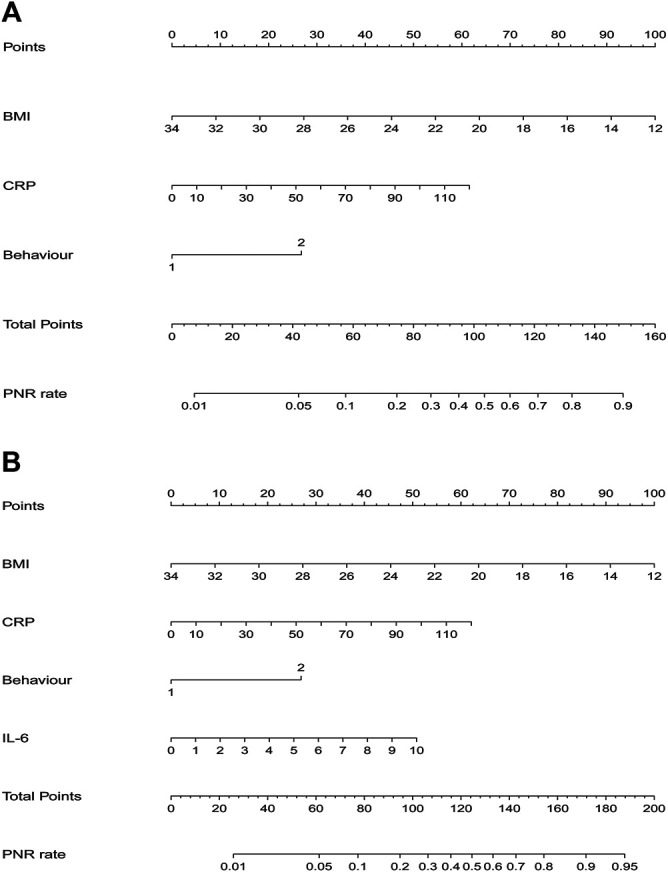
Nomograms for model 1 and model 2 were developed in the training cohort **(A)** Nomogram one for model 1, with BMI, behavior, and CRP. BMI, Body Mass Index; CRP, C-reactive protein **(B)** Nomogram two for model 2, with BMI, behavior, CRP, and IL6. IL6, interleukin-6.

Results of multiple logistic regression suggested BMI, disease behavior, CRP levels and IL-6 levels before IFX therapy were determined as predicting factors. To identify the true predictive effects of IL-6, we built a model contained IL-6 and the other one did not. The diagnostic equations of models were: logitP = 1.162–0.217* BMI +1.281 * behavior +0.025 * CRP, and logitP = 0.144–0.212* BMI +1.252 * behavior +0.024 * CRP +0.237* IL-6, respectively. The results of multivariate regression analyses are shown in Table 1. These two models are presented as nomograms, which containing these independent predictors to calculate the risk of PNR based on the total points (Figure 2).

**TABLE 1:** Multivariate regression analyses of the model 1 and model 2.

Variables	Β	Or (95% CI)	P	s.e
(a) Multivariate regression analysis including BMI, behavior, and CRP				
BMI	−0.217	0.805 (0.731–0.908)	0.000	0.061
Behavior	1.281	3.602 (1.881–6.895)	0.000	0.331
CRP	0.025	1.025 (1.009–1.041)	0.002	0.008
(b) Multivariate regression analysis including BMI, behavior, CRP, and IL-6				
BMI	−0.212	0.809 (0.716–0.914)	0.001	0.062
Behavior	1.252	3.499 (1.809–6.766)	0.000	0.336
CRP	0.024	1.024 (1.008–1.041)	0.003	0.008
IL-6	0.237	1.267 (1.041–1.541)	0.018	0.100

BMI, Body Mass Index; CRP, C-reactive protein; IL6, interleukin-6; CI, confidence interval; OR, odds ratio; s.e., standard error

### Validation of Prediction Models

**FIGURE 3:**
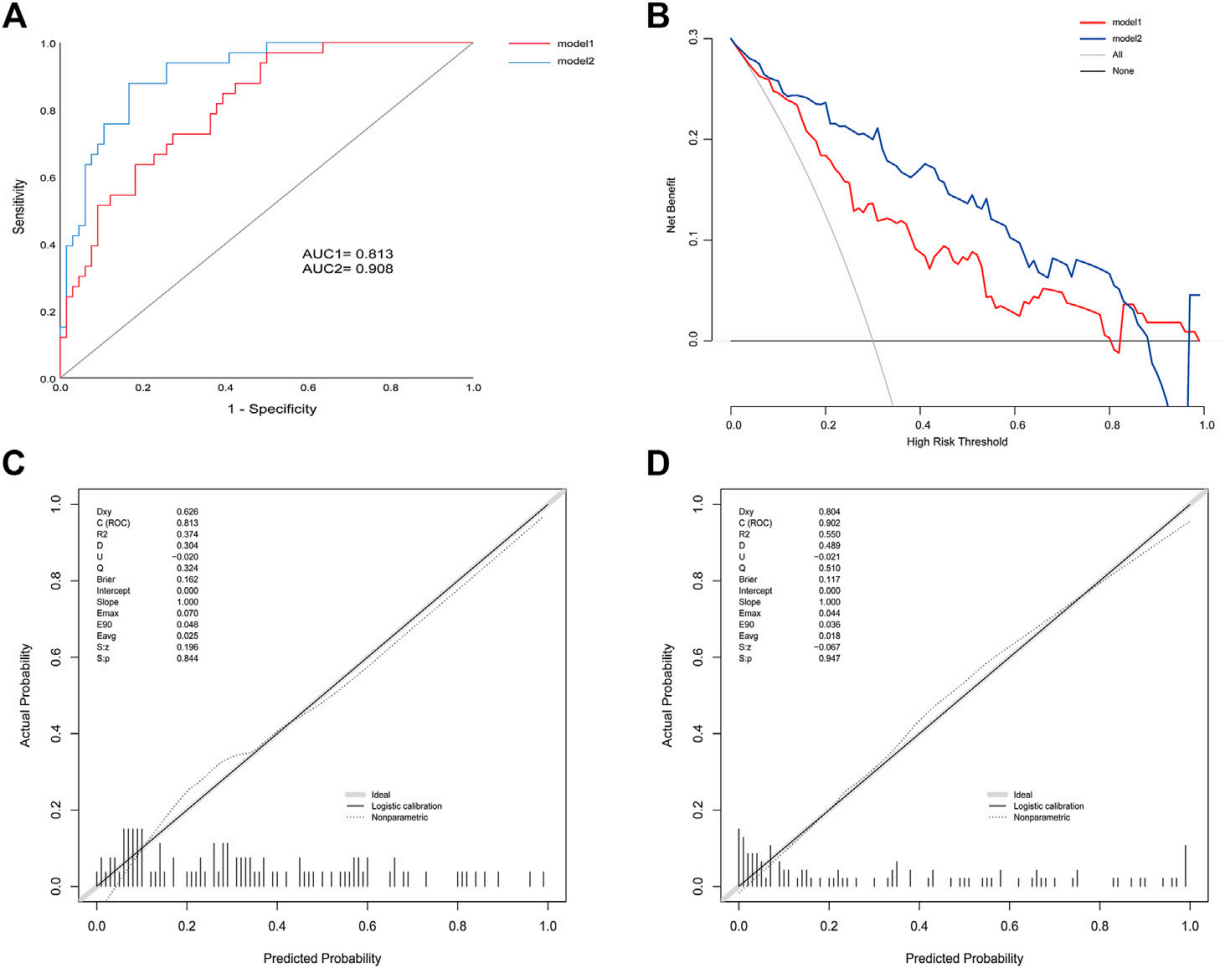
**(A)** ROC for model 1 and model 2 in the validation cohort **(B)** Calibration plots for model 1 **(C)** Calibration plots for model 2 **(D)** DCA for model 1 and model 2 in the validation cohort.

Efficacies of the two models were compared to the validation cohort. AIC was 111.3 and 99.1 of model1 and model2, respectively. ROC analysis indicated and the AUC of BMI, behavior, CRP, and IL-6 were 0.652 (95% CI: 0.540–0.764), 0.674 (95% CI: 0.563–0.785), 0.744 (95% CI: 0.646–0.842), and 0.787 (95% CI: 0.698–0.877) respectively. An AUC of 0.813 (95% CI: 0.729–0.897) in model 1 and 0.908 (95% CI: 0.851–0.966) in model 2 **(** Figure 3 **)**. The *p* value = 0.005 of DeLong’s test showed that an AUC of model 2 was significantly better than model 1. Calibration plots showed that the average differences (E aver) were 2.5 and 1.8% in model 1 and 2, and no significant differences (*P*_*1*_ = 0.844, *P*_*2*_ = 0.947) between the predicted and the calibrated probabilities. DCA showed that if the risk thresholds were between 12 and 85%, model 2 added more clinical net benefit compared to model 1. To explore additional benefits conferred by IL-6 levels, we compared IDI between models and found this improvement index was improved upon addition of IL-6 (IDI = 19%; 95% CI, 0.10–0.28; *p* < 0.000).

### Identification of Pathway and Regulatory Network of IL-6

**FIGURE 4:**
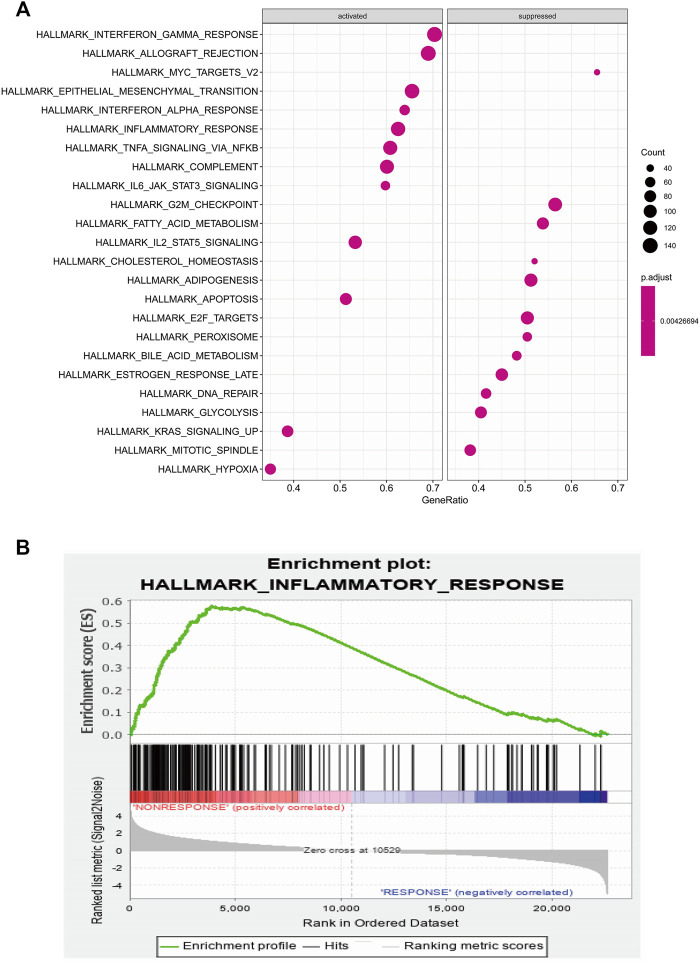
**(A)** GSEA analysis of *IL-6* and *IL-6* related genes **(B)** Inflammatory response of GSEA analysis.

**FIGURE 5:**
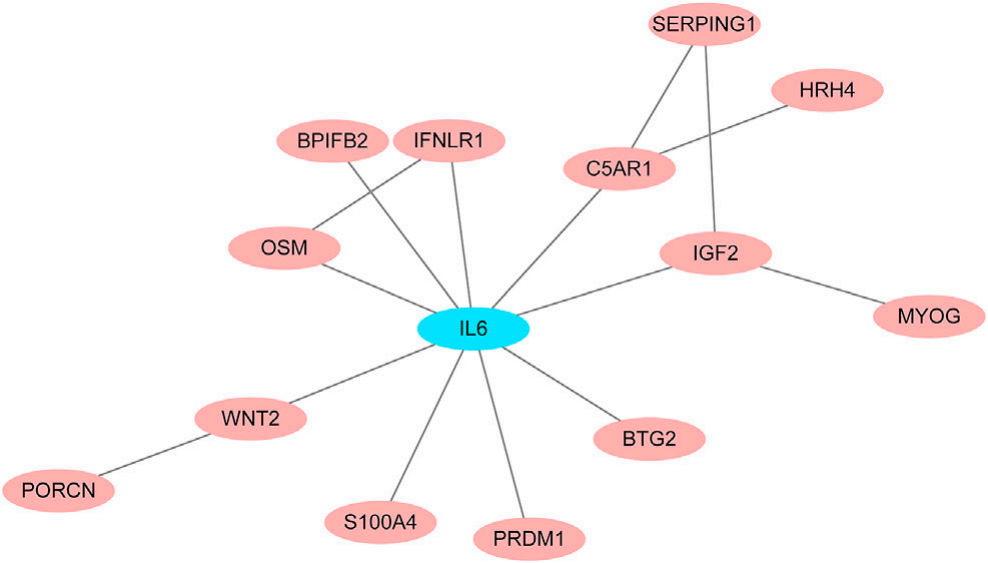
PPI networks for *IL-6* and *IL-6* related genes with the top 50 highest correlation coefficients.

**FIGURE 6:**
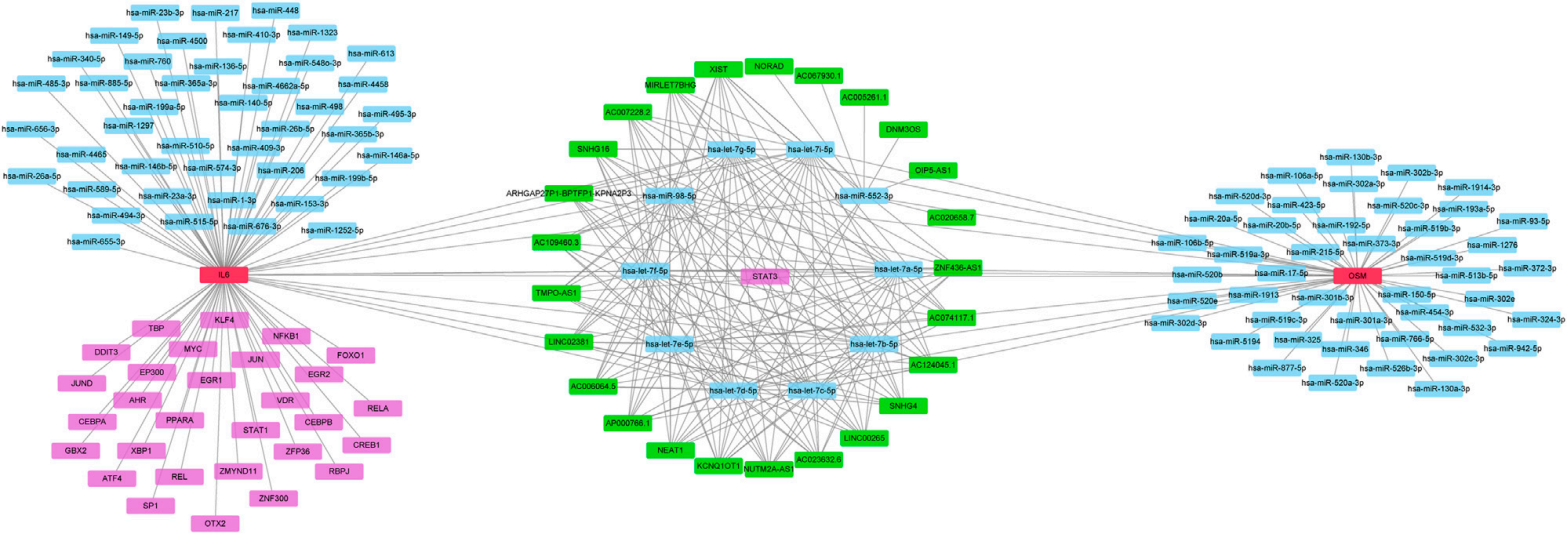
Multi-factor regulation network for *IL-6* and *OSM.*

To identify potential pathways and regulatory network of IL-6 in IFX therapy of CD, the GSE111761 dataset contained lamina propria mononuclear cells from three anti-TNF non-responders and three responders were included to identify DEGs. The samples were obtained from the patients with anti-TNF therapy for over 3 months and the Simple Endoscopic Score for Crohn’s disease (SES-CD) <5. There were 2,228 DEGs including 1,384 upregulated genes and 844 downregulated genes and IL-6 expression levels were significantly elevated in PNR patients (Supplementary Figure S2). Spearman correlation was performed to investigate the connections between *IL-6* and other DEGs. Genes with a *p* < 0.05 were selected for GSEA. Results indicated that *IL-6* and relative genes were mainly involved in inflammatory response (Figure 4; Supplementary Table S3). We selected *IL-6* related genes with the top 50 highest correlation coefficients to create PPIs, and identified five hub genes, including *IL-6, IGF2, C5AR1, IFNLR1* and *OSM* with score ≥ 4.5 based on the EPC algorithm (Figure 5). We found that the expression of Oncostatin M (*OSM*) coordinated with *IL-6* and also belonged to the inflammatory response in GSEA. To identify potential biological mechanisms between *IL-6* and *OSM*, we found a total of 10 miRNAs, 25 lncRNAs, and 1 TF shared in common with both *IL-6* and *OSM*. Data of these two genes and their miRNAs, lncRNAs and TF were integrated into a regulatory network (Figure 6). It was suggested that *IL-6* and *OSM* may be involved in the inflammatory response through a regulation network based on common lncRNAs, miRNAs and TF.

## Discussion

Even though anti-TNF agents have shown to be effective in inducing clinical response and mucosal healing, there are still a considerable proportion of patients who do not respond (Ding et al., 2016). Previous studies have identified several predictors of PNR to IFX in CD, such as BMI, fecal calprotectin (Pavlidis et al., 2016), proinflammatory biomarkers (Billiet et al., 2017), and genetic markers (Barber et al., 2016). These studies have also established prediction models based on these factors. IL-6 is a proinflammatory cytokine and expression levels alter after IFX therapy. It is considered a good predictor of IFX response (Suzuki et al., 2015; Salvador-Martín et al., 2019). However, there are few prediction models for PNR to IFX treatment in CD. In this study, we showed that IL-6 levels before IFX therapy predicted the response to IFX treatment and alter after IFX therapy.

This retrospective study included 322 CD patients who were naive to anti-TNF therapy and both their clinical and serological data were collected. Logarithmic transformation was performed for IL-6 levels to decrease the undulation of data. Consistent with previous reports, IL-6 levels were significantly reduced after 14 weeks of IFX therapy. Univariate and multivariate regression analyses found that BMI, disease behavior, CRP and IL-6 levels before IFX therapy independently predicted the response to IFX treatment. To explore the predictive ability of IL-6, we established two prediction models, one containing BMI, disease behavior and CRP levels, and the other including BMI, disease behavior, CRP levels, and IL-6 levels. Results of AIC and ROC in the validation cohort, indicated that the goodness of fit and discrimination of model containing IL-6 was better than the model not including IL-6. And the AUC of the model incorporating IL-6 was higher than that in single factors, including BMI, disease behavior and CRP levels. The calibration curve and DCA curve demonstrated a greater consistency and clinical validity in the model containing IL-6. IDI analysis showed that incorporation of IL-6 with BMI, behavior, and CRP significantly improved the discriminatory accuracy for PNR with IDI of 19% (*p* < 0.000). Furthermore, the improvement of IDI for discriminating PNR to IFX therapy is consistent with previous studies, such as Leal et al. (Leal et al., 2015), which revealed the predictive effect of IL-6 on risk of PNR to anti-TNF therapy in CD patients. Therefore, we suggested that IL-6 played a central role in assessing the possibility of PNR to IFX therapy.

Furthermore, we analyzed GEO data to verify the predictive power of IL-6. It was found that expression of *IL-6* significantly increased in non-responders compared with responders to IFX therapy. GSEA of *IL-6* and its relative genes with high correlation coefficients showed that these genes mainly were enriched in the inflammatory response. Through the construction of PPI networks, we found that *OSM* directly connected to *IL-6* and its expression was consistent with *IL-6* in the inflammatory response. *OSM* regulates the production of proinflammatory cytokines such as IL-6 through the JAK-STAT pathway (Hermanns, 2015). Studies demonstrated that *OSM* induced intestinal inflammation, while mechanisms remained unclear (Thomas, 2017). Furthermore, *OSM* has been considered a novel biomarker to predict the efficacy of anti-TNF therapy. One study showed that *OSM* was enriched in CD mucosa and complete mucosal healing was more likely to occur in patients with low *OSM* expression levels. Notably, the expression of *OSM* was strongly correlated with PNR to IFX (West et al., 2017). Another clinical study suggested that *OSM* was an appreciable biomarker in predicting the possibility of mucosal healing compared with fecal calprotectin, which indicated *OSM* may be a predictive indicator for IFX therapy (Bertani et al., 2020b). To explore the common mechanisms of *IL-6* and *OSM* in IFX treatment, we found that they both shared 10 miRNAs, 25 lncRNAs and 1 TF, which provides potential targets and pathways research diving into deeper mechanistic studies.

Although this study developed a new prediction model based on clinical and serological data, it faced several limitations. First, as a retrospective study, we defined PNR through chart review rather than prospectively collected disease activity indices. To ensure homogeneousness of the study, all CD patients were evaluated by the same physician group over the duration of IFX treatment. Second, IL-6 levels were detected in peripheral blood rather than mucosal tissue. As a result, findings represented the state of the peripheral immune system instead of the inflamed mucosa. Furthermore, external validation is required to confirm the validity of these results in clinical practice.

## Conclusion

This study found IL-6 levels altered after IFX therapy and added IL-6 to enhance the predictive value of PNR to IFX therapy in CD bio-naïve patients. A novel prediction model was developed, including IL-6 levels combined with BMI, disease behavior and CRP levels. With this model, clinicians can estimate the risk of PNR to IFX therapy and select the optimal treatment for individual patients. Furthermore, this study also constructed a multi-factor regulation network of *IL-6* and its relative gene *OSM*, providing stronger direction for exploring the predictive and therapeutic targets of IFX treatment in CD. Further investigation is needed to determine the association between IL-6 from either peripheral or inflamed mucosal and anti-TNF treatment responses.

## Data Availability

The raw data supporting the conclusions of this article will be made available by the authors, without undue reservation.
