# Supplementary material for: Development and Validation of an Interleukin-6 Nomogram to Predict Primary Non-response to Infliximab in Crohn’s Disease Patients
Source: Front Pharmacol. 2021 Apr 14;12:654985. doi: 10.3389/fphar.2021.654985 (PMC8112246; doi:10.3389/fphar.2021.654985)
Supplement: Supplementary file 1 [file Image1.tif]

Frontiers | Development and Validation of an Interleukin-6 Nomogram to Predict Primary Non-response to Infliximab in Crohn's Disease Patients | Pharmacology


- About
- Journals
- Research Topics
- Articles
- More

Submit

My Frontiers

Office

- TSOF
  - TSOF
  - Article Production

Typesetter 3

frontiersproduction@tnq.co.in

- Profile
- Settings & Privacy
- Help Center
- Logout

Submit

**Impact Factor 4.225** | **CiteScore 5.0**More on impact ›

|  |  |
| --- | --- |
| Frontiers in Pharmacology | Gastrointestinal and Hepatic Pharmacology |

Toggle navigation


Section


- (current)Section
- About
- Articles
- Research topics
- For authors 
  - Why submit?
  - Fees
  - Article types
  - Author guidelines
  - Review guidelines
  - Submission checklist
  - Contact editorial office
  - Submit your manuscript
- Editorial board

- *Article alerts*

##### This article is part of the Research Topic

Inflammation and Fibrosis in the Gastrointestinal Tract and Liver: Mechanisms and Targets
View all
14
Articles

Articles


**Suggest a Research Topic >**

- 34
  total views

 View Article Impact

**Suggest a Research Topic >**

##### SHARE ON

- Facebook

  0
- Twitter

  0
- LinkedIn

  0
- AddThis

  New


## Original Research ARTICLE

Front. Pharmacol.
| doi: 10.3389/fphar.2021.654985

# Development and Validation of an Interleukin-6 Nomogram to Predict Primary Non-response to Infliximab in Crohn's Disease Patients Provisionally accepted The final, formatted version of the article will be published soon. **Notify me**

Yueying Chen1, 
Hanyang Li1, 
Qi Feng1 and 
 Jun Shen2\*

- 1Renji Hospital, School of Medicine, Shanghai JiaoTong University, China
- 2Shanghai Jiao Tong University, China

Background: The primary nonresponse (PNR) rate of Infliximab (IFX) varies from 20% to 46% for the treatment of Crohn’s disease (CD). Detected PNR reduces the improper use of specific treatments. To date, there is hardly any knowledge regarding early markers of PNR. The aim of this study was to evaluate the role of Interleukin-6 (IL-6) as an early predictor of PNR of IFX for the treatment of CD.   
Methods: We enrolled 322 bio-naïve patients diagnosed with CD from January 2016 to May 2020. Primary response was determined at week 14. Multivariable logistic regression was used to construct prediction models. Area under the curve (AUC), calibration and decision curves analyses (DCA) were assessed in the validation cohort. GEO data were analyzed to identify potential mechanisms of IL-6 in IFX therapy for CD.  
Results: PNR occurred in 31.06% (100 of 322) patients who were assessable at week 14. IL-6 levels significantly decreased after IFX therapy (P < 0.001). The validation model containing IL-6 presented enhanced discrimination with an AUC of 0.908 and high calibration. Decision curve analysis (DCA) indicated that the model added extra predictive value. GEO data confirmed the IL-6 levels were increased in the PNR group and IL-6-related differently expressed genes (DEGs) were enriched in the inflammatory response.  
Conclusion: We concluded that IL-6 may be used as a predictive factor to assess the risk of PNR to IFX therapy.

Keywords: 
Interleukin-6, primary non-response, Crohn ‘s disease, infliximab, bioinfomatics

Received: 18 Jan 2021;
Accepted: 16 Mar 2021.

Copyright: © 2021 Chen, Li, Feng and Shen. This is an open-access article distributed under the terms of the Creative Commons Attribution License (CC BY). The use, distribution or reproduction in other forums is permitted, provided the original author(s) and the copyright owner(s) are credited and that the original publication in this journal is cited, in accordance with accepted academic practice. No use, distribution or reproduction is permitted which does not comply with these terms.

\* Correspondence: 
Mx. Jun Shen, Shanghai Jiao Tong University, Shanghai, China, shenjun\_renji@163.com

Write a comment...

Add

##### COMMENTARY

##### ORIGINAL ARTICLE

##### People also looked at

## Jianpi Jieyu Decoction, an empirical herbal formula, exerts psychotropic effects in association with modulation of gut microbial diversity and GABA activity

Liu Lan Ying, Zhilu Zou, Jiangwei Yang, Xiaoqi Li, Boran Zhu, Hailou Zhang, Yan Sun, Yuxuan Zhang and Wei Wang

**Suggest a Research Topic >**

×

#### Supplementary Material

  

There is no supplementary material currently available for this article

Loading supplemental data...

  

|  | File Name |  |
| --- | --- | --- |
|  | Table 1.docx |  |
|  | Table 2.docx |  |
|  | Table 3.docx |  |
|  | Image 1.TIF |  |
|  | Image 2.TIF |  |

  

Close

- About Frontiers
- Institutional Membership
- Books
- News
- Frontiers' social media
- Contact
- Careers
- Submit
- Newsletter
- Help Center
- Terms & Conditions
- Privacy Policy

© 2007 - 2021 Frontiers Media S.A. All Rights Reserved

### Privacy Preference Center

Our website uses cookies that are necessary for its operation. Additional cookies are only used with your consent. These cookies are used to store and access information such as the characteristics of your device as well as certain personal data (IP address, navigation usage, geolocation data) and we process them to analyse the traffic on our website in order to provide you a better user experience, evaluate the efficiency of our communications and to personalise content to your interests. Some cookies are placed by third-party companies with which we work to deliver relevant ads on social media and the internet. Click on the different categories' headings to change your cookie preferences. Click on "More Information" if you wish to learn more about how data is collected and shared.
More information

### Manage Consent Preferences

#### Strictly Necessary Cookies

Always Active

These cookies are necessary for the website to function and cannot be switched off in our systems. They are usually only set in response to actions made by you which amount to a request for services, such as setting your privacy preferences, logging in or filling in forms. You can set your browser to block or alert you about these cookies, but some parts of the site will not then work. These cookies do not store any personally identifiable information.

#### Analytics Cookies

Analytics Cookies

These cookies allow us to count visits and traffic sources so we can measure and improve the performance of our site. They help us analyse which pages are the most and least popular and see how visitors move around the site.    All information these cookies collect is aggregated and therefore anonymous.

#### Functional Cookies

Functional Cookies

These cookies enable the website to provide enhanced functionality and personalisation. They may be set by us or by third party providers whose services we have added to our pages. If you do not allow these cookies then some or all of these services may not function properly.

#### Advertising Cookies

Advertising Cookies

These cookies may be set through our site by our advertising partners. They may be used by those companies to build a profile of your interests and show you relevant adverts on other sites.    They do not store directly personal information, but are based on uniquely identifying your browser and internet device. If you do not allow these cookies, you will experience less targeted advertising.

### Back Button Performance Cookies

Vendor Search  Search Icon

Filter Icon

Clear

checkbox label label

Apply Cancel

Consent Leg.Interest

checkbox label label

checkbox label label

checkbox label label

Confirm My Choices
