# Supplementary material for: Development and Validation of an Interleukin-6 Nomogram to Predict Primary Non-response to Infliximab in Crohn’s Disease Patients
Source: Front Pharmacol. 2021 Apr 14;12:654985. doi: 10.3389/fphar.2021.654985 (PMC8112246; doi:10.3389/fphar.2021.654985)
Supplement: Supplementary file 3 [file table1.docx]

| Characteristics | Training cohort （n=223） | Validation cohort  (n=99) |
| --- | --- | --- |
| primary non-response/ response (n) | 67/156 | 33/66 |
| Gender (male/female, n) | 170/53 | 75/24 |
| Age (median, IQR), year | 28(22-35) | 27(21-33) |
| BMI (median, IQR) | 19.8 (18-21.61) | 19.5(17.36-21.5) |
| Smoking (yes/no, n) | 10/213 | 4/95 |
| Surgery (yes/no, n) | 79/144 | 38/61 |
| Duration (median, IQR), month | 36(24-54) | 36(24-54) |
| Location (n) |  |  |
| L1 | 76 | 31 |
| L2 | 30 | 17 |
| L3 | 109 | 48 |
| L4 | 8 | 3 |
| Behavior (B1/B2, n) | 122/101 | 47/52 |
| perianal disease (yes/no, n) | 140/83 | 58/41 |
| HBI (mean ±sd) | 7.62 (±2.15) | 7.49(±2.057) |

Supplementary Table 1. Comparison of Demographics Between Training and Validation cohort

BMI, Body Mass Index; L1, terminal ileum; L2, colon; L3, ileocolon; L4, upper gastrointestinal; B1,nonstricturing nonpenetrating; B2, stricturing and/or penetrating; HBI, Harvey Bradshaw indices; sd, standard deviation; IQR, interquartile range.
